# Maintenance treatment with trofosfamide in patients with advanced soft tissue sarcoma – a retrospective single-centre analysis

**DOI:** 10.2340/1651-226X.2025.42356

**Published:** 2025-01-15

**Authors:** Anton Burkhard-Meier, Vera Valerie Rechenauer, Luc M. Berclaz, Vindi Jurinovic, Markus Albertsmeier, Hans Roland Dürr, Sinan E. Güler, Michael Hoberger, Alexander Klein, Thomas Knösel, Wolfgang G. Kunz, Nina-Sophie Schmidt-Hegemann, Michael von Bergwelt-Baildon, Lars H. Lindner, Dorit Di Gioia

**Affiliations:** a Comprehensive Cancer Center Munich and Department of Medicine III, University Hospital, LMU Munich, Munich, Germany; b Bavarian Cancer Research Center (BZKF), Munich, Germany; c German Cancer Consortium (DKTK), Partner Site Munich, Munich, Germany; d Institute for Medical Information Processing, Biometry, and Epidemiology, University Hospital, LMU Munich, Munich, Germany; e Department of General, Visceral and Transplantation Surgery, University Hospital, LMU Munich, Munich, Germany; f Department of Orthopedics and Trauma Surgery, University Hospital, LMU Munich, Munich, Germany; g Institute of Pathology, University Hospital, LMU Munich, Munich, Germany; h Department of Radiology, University Hospital, LMU Munich, Munich, Germany; i Department of Radiation Oncology, University Hospital, LMU Munich, Munich, Germany

**Keywords:** Metastasis, soft tissue sarcoma, trofosfamide, systemic therapy, maintenance

## Abstract

**Background:**

The prognosis of patients with advanced soft tissue sarcoma (STS) remains dismal. Trofosfamide (TRO) has been proposed as a well-tolerated oral maintenance therapy. This retrospective analysis aims to determine the value of this therapy.

**Methods:**

Fifty-nine patients with advanced STS who received TRO maintenance therapy between 2016 and 2022 were reviewed and analysed regarding clinical parameters and outcomes.

**Results:**

The median age was 48 years; the most common histological subtype was synovial sarcoma (*n* = 22, 37%), and 71% of patients (*n* = 42) presented with metastatic disease. No radiological evidence of disease (NED) before the start of maintenance was reported in 36% of patients (*n* = 21). The median follow-up was 38.2 months with a median maintenance duration of 9.0 months. The median event-free survival (EFS) and overall survival (OS) were 9.5 and 33.2 months, respectively. In metastatic patients achieving NED before the initiation of TRO, the median EFS was 29.4 months, while the median OS was not reached. In metastatic patients with anthracycline + ifosfamide (AI) as first-line induction therapy without prior metastasis-directed local therapy, the median EFS and OS from the start of AI were 13.9 and 26.8 months, respectively. Multivariate analysis of the overall cohort demonstrated that NED before the start of maintenance was significantly associated with a prolonged EFS (*p* = 0.024, hazard ratio [HR] = 0.26), and G2 histology correlated with longer OS (*p* = 0.030, HR = 0.16, reference: G3).

**Interpretation:**

Oral maintenance therapy with TRO appears to improve outcomes in patients with advanced STS. Metastatic patients who achieve NED through prior metastasectomy may particularly benefit from TRO maintenance.

## Introduction

Soft tissue sarcomas (STS) are rare mesenchymal malignancies, accounting for approximately 1% of all adult cancers [1]. Up to half of patients with STS develop metastases during the course of their disease, resulting in a poor prognosis with a median overall survival (OS) of 12–24 months [2–5]. To reduce the risk of distant metastasis, localised high-risk (≥5 cm, high-grade, deep to the fascia) STS should be considered for neoadjuvant systemic therapy, which may be combined with regional hyperthermia (RHT) if available [6, 7]. For metastatic STS, anthracycline-based chemotherapy remains the standard treatment [8]. While the combination with ifosfamide led to a higher response rate in this setting, it has not demonstrated an OS benefit [9]. Local therapies (LT), such as surgical metastasectomy and stereotactic radiotherapy (RT), are associated with improved survival in patients with limited metastatic disease [10, 11]. Furthermore, maintenance therapies are increasingly recognised as an important component in the treatment of advanced solid tumours and have been incorporated into recent phase III sarcoma trials, such as leiomyosarcoma (LMS)-04 and iEuroEwing [12, 13].

Trofosfamide (TRO) is an oral alkylating agent belonging to the oxazaphosphorine class, such as ifosfamide and cyclophosphamide [14]. Although TRO is approved only for lymphoma treatment in Europe [15], it has been used in STS for many years [16–18]. Its off-label use includes maintenance therapy following a response to prior chemotherapy or as an alternative to anthracyclines in elderly patients. Reichardt et al. retrospectively analysed 49 patients with locally advanced or metastatic STS or bone sarcoma who received TRO maintenance following systemic induction therapy that achieved partial response (PR) or stable disease (SD). This study demonstrated a survival benefit compared to historical cohorts [19]. Hartmann et al. conducted a randomised phase II trial in patients over 60 years with metastatic STS, comparing TRO to doxorubicin as first-line treatment. The primary endpoint of a 6-month progression-free rate was exceeded (35.9% vs. 27.6%), and TRO demonstrated a favourable toxicity profile, predominantly low-grade dyspnoea, fatigue and well-manageable haematotoxicity [20]. Previous studies on TRO in STS consistently report very good tolerability [16–20].

At our high-volume sarcoma centre, TRO is commonly used as a maintenance therapy in STS patients, based on the findings of Reichardt et al. [19]. Given its favourable toxicity profile, the use of TRO has been extended to patients with metastatic or locally advanced high-risk STS following surgical resection with no radiological evidence of disease (NED). However, to date, no studies have evaluated the efficacy of TRO in this patient population. This study aims to evaluate the efficacy and toxicity of TRO as a maintenance therapy for STS and to identify the patient subgroups most likely to benefit from this regimen.

## Materials and methods

### Patient selection and data extraction

Eligible patients (age ≥18 years) had pathologically confirmed locally advanced or metastatic STS and received TRO as maintenance therapy between January 2016 and December 2022. Patients who received TRO as an alternative regimen to anthracycline-based chemotherapy, as described by Hartmann et al., were excluded [20]. Clinical, pathological and outcomes data were extracted from our prospectively maintained sarcoma database. The updated World Health Organization (WHO) tumour classification system for soft tissue and bone tumours and the French grading system were applied [21, 22]. For ‘non-gradable’ histologies, a grade was created based on the chemosensitivity of the respective subtype (e.g. synovial sarcoma: grade 3). Dates of death were determined with the help of the Cancer Registry of Bavaria. Due to the retrospective and anonymised nature of the study, informed consent was not required. The Internal Review Board and the Ethical Review Committee at the Ludwig Maximilians University (LMU) Hospital, Munich, Germany, approved the protocol of this research project (Protocol Nr. 23-0618).

### Imaging analysis

Computed tomography (CT), magnetic resonance (MR) and positron emission tomography with 18-fluorodeoxyglucose (FDG-PET-CT) images were reviewed by a radiologist with subspecialty training in oncological imaging (WGK). The Response evaluation criteria in solid tumours (RECIST) v1.1 were applied to evaluate the efficacy of systemic induction therapy, TRO therapy and subsequent systemic therapy. NED was defined as the absence of radiologically visible tumour at the start of TRO.

### Treatment

Patients received oral TRO at a target dose of 150–200 mg/day continuously, according to the study protocol published by Hartmann and colleagues [23]. In contrast to this protocol and based on our institution’s experiences, a daily starting dose of 100 mg was chosen and escalated after 1 week depending on tolerability. Weekly blood counts were performed through the patients’ general practitioners. Treatment was interrupted in the case of grade III haematological toxicity (leukocytopenia <2 G/L, haemoglobin <8 g/dL or thrombocytopenia <50 G/L). Following recovery to at least grade II toxicity, treatment was resumed at a reduced dose (50 mg/day less). Therapy was discontinued upon severe toxicity or significant disease progression. Additionally, for patients receiving treatment beyond 24 months, a risk‑benefit assessment considering factors such as tumour burden and tolerability was conducted, and the continuation of treatment was discussed with the patient. Toxicity was measured according to CTCAE v.5 [24]. Event-free survival (EFS) was measured from the start of maintenance until the next tumour progression, recurrence or death. OS was calculated from the start of maintenance until death. EFS2 was defined as the duration from the start of maintenance treatment until the second tumour progression, recurrence or death. Additional analyses included measuring EFS and OS from the start of induction therapy and subsequent systemic therapy, respectively. The starting points are consistently clarified in the results section. Systemic induction therapy was defined as prior systemic therapy without tumour progression before the start of maintenance therapy. The end of follow up was February 29, 2024.

### Statistical analysis

OS and EFS were analysed using Cox proportional hazards regression. The results with a *p*-value of ≤0.05 were considered statistically significant. Statistical analysis was performed using R software version 4.0.3 (R Foundation for Statistical Computing, Vienna, Austria).

## Results

### Patient characteristics

The clinicopathologic characteristics of the study cohort are summarised in Table 1. Between January 2016 and December 2022, 59 patients initiated TRO maintenance therapy at our institution. The median age at initial diagnosis was 48 years (range 18–74 years), with 66% of patients (*n* = 39) being female. At the start of TRO, 71% (*n* = 42) presented with metastatic disease, while 29% (*n* = 17) had locally advanced disease.

**Table 1:** Patient characteristics.

Factor	Strata	*n*	%
Total		59	100
Sex	Male	20	34
	Female	39	66
Age at first diagnosis (years)	<60	44	75
	≥60	15	25
Histological subtype	Synovial sarcoma	22	37
	Undifferentiated pleomorphic sarcoma	6	10
	Liposarcoma, myxoid	6	10
	Liposarcoma, dedifferentiated	4	7
	Myxofibrosarcoma	3	3
	Leiomyosarcoma, non-uterine	3	5
	Leiomyosarcoma, uterine	2	3
	MPNST	2	3
	Angiosarcoma	2	3
	Undifferentiated uterine sarcoma	2	3
	Endometrial stroma sarcoma (high grade)	2	3
	Others (max. one patient)*	5	8
Localisation of primary tumour	Extremities	13	22
	Visceral/retroperitoneal	17	29
	Trunk	13	22
	Uterus	7	12
	Head/Neck	1	2
	Other or unclear	8	14
Histology grade at first diagnosis	G1	2	3
	G2	20	34
	G3	37	63
AJCC stage at first diagnosis	IB	1	2
	II	7	12
	IIIA	20	34
	IIIB	12	20
	IV	19	32
Disease stage at start of TRO	Locally advanced	17	29
	Metastatic	42	71
Radiological evidence of disease at start of TRO	Yes	38	64
	No	21	36

MPNST: Malignant peripheral nerve sheath tumour; AJCC: American Joint Committee on Cancer; TRO: Trofosfamide.

* Supplementary material 1.

In the study cohort, 36% of patients (*n* = 21) had NED at the time of TRO initiation. Among the NED subgroup, 76% (*n* = 16) underwent incomplete resection of primary tumour or metastases (R1/R2) before the start of TRO, and 14% (*n* = 3) had prior peritoneal or pleural sarcomatosis. Two NED patients had a complete resection (R0) with no suspected residual disease but were considered to be at very high risk of recurrence (short disease-free interval, high tumour burden). The median number of prior locally treated metastases in the metastatic NED subgroup was 2 (range 1–19).

Indications for TRO maintenance were determined by our institution’s multidisciplinary tumour board. The most commonly reported decision-making factors were response or stabilisation with induction therapy (*n* = 33, 56%), lack of LT options (*n* = 13, 21%), high risk of recurrence (*n* = 11, 19%), residual tumour (R1/R2 situation or suspected metastatic disease; *n* = 9, 14%) and high tumour burden prior to metastasectomy (*n* = 7, 12%). Some tumour board decisions involved multiple indication factors, leading to a total count exceeding the number of patients. However, not all indication factors were consistently documented in the tumour board decision reports.

### Induction treatment and further prior therapies

Among the analysed patients, 83% (*n* = 49) received systemic induction therapy, and 64% (*n* = 38) underwent additional LT. Ten patients (17%) were only treated locally at the respective tumour diagnosis, progression or recurrence prior to TRO. Anthracycline + ifosfamide (AI) ± RHT was the most common induction regimen (80%, *n* = 39). RECIST responses among patients with systemic induction therapy included PR in 43% (*n* = 21) and SD in 47% (*n* = 23). Additionally, one patient achieved a complete response (CR), while another experienced progressive disease (PD) according to RECIST criteria, but showed a response based on Choi criteria [25]). Details on prior treatment are provided in Table 2.

**Table 2:** Prior therapies.

Factor	Strata	*n*	%
Number of prior systemic therapies	0	2	3
	1	43	75
	2	12	21
	≥3	2	3
Systemic induction therapy	Yes	49	83
	No	10	17
Response to systemic induction therapy (RECIST)	CR	1	2
	PR	21	43
	SD	23	47
	PD*	1	2
	N/A	3	6
Induction regimen	AI (+ RHT)	39 (23)	80 (47)
	HDI	6	12
	AD (+ RHT)	3 (2)	6 (4)
	ICE + RHT	1	2
LT before start of TRO	Yes, metastases	15	25
	Yes, primary tumour	25	42
	Yes, primary tumour and metastases	8	14
	No	11	19
LT of primary tumour before start of TRO	Yes, surgery	22	37
	Yes, surgery + RT	8	14
	Yes, RT	3	5
	No	26	44
LT of metastases before start of TRO	Yes, surgery	14	24
	Yes, surgery + RT	3	5
	Yes, RT	6	10
	No	36	61

AI: Anthracycline + ifosfamide; RECIST: Response Evaluation Criteria in Solid Tumours; CR: Complete response; PR: Partial response; SD: Stable disease; PD: Progressive disease; N/A: Not applicable; RHT: Regional hyperthermia; AI: Anthracycline + ifosfamide; HDI: High-dose ifosfamide; AD: Anthracycline + dacarbazine; ICE: Ifosfamide + carboplatin + etoposide; LT: Local therapy; TRO: Trofosfamide; RT: Radiotherapy.

* Response according to Choi criteria.

### Treatment and toxicity

The median duration of TRO therapy was 9.0 months (95% CI 5.6–12.1 months). Treatment was discontinued in 81% (*n* = 48) due to tumour progression, in 3% (*n* = 2) due to toxicity and in 5% (*n* = 3) following a risk‑benefit assessment after 2 years. Of the latter, two remained disease-free until the end of follow-up (8 and 18 months after stopping TRO), while one developed tumour progression after 10 months. Six patients continued TRO until the end of follow-up.

In 78% of patients (*n* = 46), a maximum dose of 200 mg/day was applied after dose escalation, as specified above. Due to limited tolerability, the maximum doses were 150 mg/day and 100 mg/day in 20% (*n* = 12) and 2% (*n* = 1) of patients, respectively. Dose reductions and/or treatment interruptions occurred in 31% (*n* = 18) due to toxicity.

Haematological toxicities were evaluated in 92% of patients (*n* = 54). Anaemia, leukocytopenia and thrombocytopenia of grade I–II were reported in 65% (*n* = 35), 56% (*n* = 30) and 13% (*n* = 7) of patients, respectively. Anaemia, leukocytopenia and thrombocytopenia of grade III were documented in 4% (*n* = 2), 15% (*n* = 8) and 2% (*n* = 1), respectively. No grade IV haematotoxicity was observed. Other possibly related toxicities included depressive mood (grade II, *n* = 2), fatigue (grade II, *n* = 3), dizziness (grade II, *n* = 1), dyspnoea (grade I, *n* = 1), chronic kidney disease (grade III, *n* = 1) and occlusal dysaesthesia (grade II, *n* = 1). No secondary malignancies were reported in the follow-up.

### Efficacy

**Figure 1:**
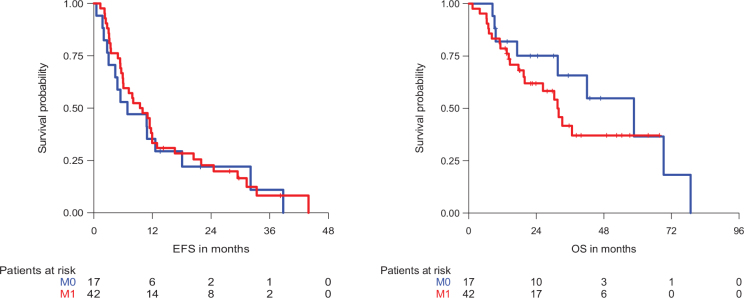
Event-free survival (EFS) and overall survival (OS) of patients with trofosfamide (TRO) maintenance therapy according to disease stage. M0: Locally advanced, M1: Metastatic.

The median follow-up was 38.2 months. The median EFS for patients with TRO maintenance was 9.5 months, while the median OS was 33.2 months, corresponding to 1- and 2-year OS rates of 79.6% (95% confidence interval [CI] 69.9–90.6%) and 65.8% (95% CI 54.4–79.8%), respectively. The EFS and OS of patients with metastatic and locally advanced disease is illustrated in Figure 1.

Among the metastatic patients with NED at the start of TRO (*n* = 11), the median EFS was 29.4 months, while the median OS was not reached. Metastatic patients with evidence of disease (i.e. radiologically visible tumour; *n* = 31) had a median EFS and OS of 7.2 and 26.3 months, respectively.

The patients with locally advanced disease and NED at the start of TRO (*n* = 10) presented with a median EFS and OS of 8.1 and 58.6 months, respectively. The median EFS and OS for patients with locally advanced disease and evidence of disease (*n* = 7) were 6.9 and 69.2 months, respectively.

In all patients with AI ± RHT as a first-line treatment (*n* = 39), the median EFS and OS from the start of AI, including TRO maintenance, were 16.4 and 39.0 months, respectively. In patients with AI without RHT (*n* = 16), the median EFS and OS were 17.8 and 34.4 months, respectively. In metastatic patients with AI ± RHT (in the case of accessible oligometastasis) as a first-line treatment and no further metastasis-directed LT (*n* = 14), the median EFS and OS from the start of AI were 13.9 and 26.8 months, respectively.

Response to TRO according to RECIST 1.1 could be evaluated in 38 patients (64%). One patient showed a PR, while SD was achieved in 24 patients (63%).

### Prognostic factors for outcome after TRO maintenance therapy

Univariate analysis identified NED and female sex as positive prognostic factors for EFS (*p* = 0.028, hazard ratio [HR] = 0.50 and *p* = 0.026, HR = 0.52, respectively). For OS, female sex (*p* = 0.022, HR = 0.42) and primary tumour size ≥5 cm (*p* = 0.033, HR = 2.77) were significant factors. Grading, primary tumour localisation, metastatic stage before the start of TRO, response to ifosfamide-containing therapy or induction therapy, prior systemic therapies and dose reduction/treatment interruption did not significantly influence survival (Supplementary material 2).

Multivariate analysis revealed NED as a positive prognostic factor for EFS, while G2 histology correlated with improved OS (Table 3).

**Table 3:** Prognostic factors for event-free survival and overall survival after maintenance therapy with trofosfamide, multivariate analysis.

Factor	Strata	EFS		OS	
		*P*	HR (95% CI)	*P*	HR (95% CI)
Age	≤60 vs. >60	*0.063*	3.38 (0.93–12.26)	0.48	1.85 (0.33–10.30)
Sex	Female vs. male	0.88	0.93 (0.33–2.57)	0.47	0.59 (0.14–2.48)
Histological subtype	Rest vs. SySa	0.77	1.22 (0.33–4.54)	0.82	0.83 (0.17–4.18)
Grading	G2 vs. G3	0.30	0.56 (0.19–1.67)	**0.030**	0.16 (0.031–0.84)
Disease stage at the start of TRO	Metastatic vs. Locally advanced	0.30	0.56 (0.19–1.67)	0.11	3.56 (0.75–16.95)
NED at the start of TRO	Yes vs. No	**0.024**	0.26 (0.079–0.83)	0.21	0.43 (0.11–1.60)

EFS: Event-free survival, OS: Overall survival, SySa: Synovial sarcoma, TRO: Trofosfamide, NED: No radiological evidence of disease, HR: Hazard ratio, CI: Confidence interval. Bold values indicate statistical significance at the *p*<0.05 level. Italic values indicate *p*<0.1.

### Subsequent treatments

66% (*n* = 39) of patients received subsequent systemic therapies, most commonly trabectedin (31%, *n* = 12), pazopanib (23%, *n* = 9), gemcitabine/docetaxel (18%, *n* = 7) and high-dose ifosfamide (18%, *n* = 7).

The median EFS from the start of subsequent systemic therapy was 5.0 months. The median EFS of patients with trabectedin, pazopanib, gemcitabine/docetaxel and high-dose ifosfamide were 3.8, 6.9, 2.1 and 3.8 months, respectively.

The median EFS2 (start of TRO until second progression or death) was 17.7 months. The median EFS2 of patients with trabectedin, pazopanib, gemcitabine/docetaxel and high-dose ifosfamide as following treatment regimens were 9.6, 18.1, 11.7 and 21.6 months. Multivariate analysis did not reveal a significant difference in EFS2 with regard to the subsequent treatment regimen (Supplementary material 2).

## Discussion

This study analysed patients with advanced STS who received TRO maintenance therapy between 2016 and 2022. While the activity of TRO in STS has been recognised for many years, the optimal timing of its use and prognostic factors remain undefined. With the increasing incorporation of maintenance therapies in the treatment of solid tumours, an updated analysis of TRO maintenance in STS is required.

In our cohort, the median EFS and OS were 9.5 and 33.2 months, respectively. The reported toxicity was mainly low grade and well-manageable being in line with previous studies [16–20].

Reichardt et al. reported a median progression-free survival (PFS) and OS of 7 and 14 months, respectively, in their study of STS (*n* = 37) and bone sarcoma (*n* = 12) patients. Notably, all patients in their study presented with residual macroscopic tumour (no prior LT, responses to prior induction therapy: PR in *n* = 20 and SD in *n* = 29). Additionally, their cohort included more heavily pre-treated (≥2 prior systemic therapies: 47% vs. 24%) but fewer patients with locally advanced disease (18% vs. 29%) compared to our study. As their study was conducted over 20 years ago, fewer local ablative and systemic therapy options were available than in our study period [19].

Interpreting the efficacy of TRO maintenance is challenging due to the heterogeneity of the cohort and the lack of a control group. Moreover, our findings are strongly influenced by selection bias, as the indication for TRO maintenance was determined in our multidisciplinary tumour board. The most common decision-making factors included response to induction therapy and a high risk of recurrence.

This study included patients in both metastatic and locally advanced stages and those with evidence of disease and NED at the start of TRO. Differentiating between the treatment benefits of an adjuvant and a consolidation treatment is important. In our cohort, 90% of patients with NED had undergone incomplete resection or experienced prior sarcomatosis, suggesting the presence of at least microscopic residual tumour. The remaining two patients with NED underwent systemic induction therapy and LT and were considered to be at a very high risk of recurrence. Nonetheless, the survival outcomes of this study should be analysed separately for the respective subgroups.

The outcome of these subgroups might be compared with historical outcomes of similar patients without maintenance treatment. In our cohort, the most commonly used induction therapy was AI. In 59%, the regimen was complemented by RHT according to a phase III trial from our institution that demonstrated a significant survival benefit from the addition of RHT to neoadjuvant chemotherapy in patients with localised high-risk STS [7]. In our study, survival rates were comparable between those treated with and without RHT. The median EFS and OS from the start of first-line AI ± RHT were 16.4 and 39.0 months, respectively. The subgroup of metastatic patients who did not receive additional metastasis-directed LT had a median EFS and OS of 13.9 and 26.8 months, respectively. In a phase III trial, Judson et al. demonstrated a median PFS and OS of 7.4 and 14.3 months for AI as a first-line treatment in patients with locally advanced, unresectable or metastatic STS [9]. Our results suggest a potential survival benefit from TRO maintenance therapy following disease stabilisation with AI. However, the selection of patients with chemosensitive disease for TRO maintenance should also be considered when comparing outcomes. Additionally, RHT and RT might have positively influenced our cohort’s outcome.

In our study, 39% of patients received an additional metastasis-directed LT, which has been associated with improved survival rates in previous retrospective analyses [10, 11, 26]. In metastatic patients with NED at start of TRO (achieved by prior metastasectomy), survival was exceptionally high, with a median EFS of 29.4 months and a non-reached median OS after a median follow-up of 38.2 months. It is essential to consider the high proportion of patients with incomplete resection or pleural/peritoneal sarcomatosis in this subgroup. For pulmonary metastasectomy as the most common metastasis-directed LT in STS, 20–58% 5-year-survival rates have been reported in previous studies [27]. Current guidelines do not recommend systemic therapy in addition to metachronous metastasectomy [28]. However, whether specific patient subgroups (e.g. patients with incomplete resection) benefit from additional systemic therapy remains unclear. Our results indicate that adding TRO maintenance therapy might improve outcomes in this situation. Certainly, our findings must be interpreted with caution, as the observed long-term survival may be more reflective of the disease biology rather than the effect of TRO maintenance.

In our study, prior response to an ifosfamide-containing treatment did not correlate with a survival benefit after TRO. This suggests that TRO maintenance may also be effective in patients who did not respond to prior oxazaphosporine therapy. Notably, three patients in our cohort received AD as induction therapy. Whether response to other alkylating agents, such as dacarbazine, should be considered before initiating TRO, requires further investigation in less heterogeneous cohorts.

The European Medicines Agency (EMA) has recommended using PFS2 (start of TRO until second progression or death) as an endpoint for evaluating maintenance therapies to account for potential effects on next-line therapies [29]. EFS after subsequent therapy was additionally examined to put the outcomes into context. The median EFS from the start of subsequent systemic therapy after TRO was 5.0 months, with 24% of patients receiving subsequent therapy as a third- or further-line regimen. The median EFS of trabectedin and pazopanib are in accordance with the results of their approval-relevant trials or subgroup analyses [30–32]. The median EFS after high-dose ifosfamide and gemcitabine/docetaxel was relatively poor compared to previous relevant trials [33, 34]. However, these patients had a relatively long EFS2, indicating a prolonged stabilisation under TRO. The small number and heterogeneity of patients in these subgroups hinder drawing conclusions regarding subsequent therapy outcomes.

Our findings underscore the value of a well-tolerated oral maintenance therapy with TRO in patients with STS. Particularly in patients following high-risk metastasectomy, TRO maintenance may represent a valid strategy to reduce the risk of recurrence. These results provide hypothesis-generating evidence warranting further investigation in a randomised clinical trial.

## Supplementary Material



## Data Availability

The data that support the findings of this study are not openly available due to reasons of sensitivity and are available from the corresponding author upon reasonable request.
