# Supplementary material for: Maintenance treatment with trofosfamide in patients with advanced soft tissue sarcoma – a retrospective single-centre analysis
Source: Acta Oncol. 2025 Jan 15;64:42356. doi: 10.2340/1651-226X.2025.42356 (PMC11748174; doi:10.2340/1651-226X.2025.42356)
Supplement: Supplementary file 1 [file AO-64-42356-s1.pdf]

Supplementary material has been published as submitted. It has not been copyedited, or typeset by Acta Oncologica

Supplementary Material

1. Histological subtypes: “Others”

**Histological subtype**	**n**	**%**
Liposarcoma, pleomorphic	1	2
Rhabdomyosarcoma	1	2
Solitary fibrous tumor	1	2
Undifferentiated spindle cell sarcoma	1	2
Uterine adenosarcoma	1	2

2. Prognostic factors for event-free survival (EFS) and overall survival (OS) after maintenance therapy with trofosfamide, univariate analysis

		EFS		OS	
**Factor**	**Strata**	**p-value**	**HR (95% CI)**	**p-value**	**HR (95% CI)**
Age (years)	≤60 vs. >60	0.90	0.96 (0.50-1.83)	0.19	0.57 (0.24-1.33)
Sex	Female vs. male	**0.026**	0.52 (0.29-0.92)	**0.022**	0.42 (0.20-0.88)
Histological subtype	UPS vs. SySa	0.15	2.09 (0.76-5.77)	0.54	1.49 (0.41-5.47)
	LPS vs. SySa	0.51	1.31 (0.58-2.96)	0.89	0.92 (0.29-2.95)
	LMS vs. SySa	0.97	0.98 (0.36-2.67)	0.25	0.30 (0.04-2.37)
	Others vs. SySa	0.14	1.70 (0.84-3.44)	0.27	1.64 (0.68-3.97)
Grading	G2 vs. G3	0.41	0.78 (0.44-1.40)	*0.086*	0.48 (0.21-1.11)
Primary tumor localization	Extremities vs. trunk	0.84	1.09 (0.47-2.50)	0.30	1.74 (0.62-4.92)
	Visceral/ retroperitoneal vs. trunk	0.46	0.75 (0.34-1.62)	0.69	0.81 (0.28-2.33)
	Others vs. trunk	0.46	0.74 (0.34-1.62)	0.71	0.82 (0.29-2.35)
Primary tumor size	≥5 vs. <5 cm	0.56	0.84 (0.47-1.50)	**0.033**	2.77 (1.09-7.05)
Distant metastasis before start of TRO	Lung only vs. No	0.71	0.87 (0.43-1.77)	0.22	1.84 (0.70-4.81)
	Other single site only vs. No	0.90	0.93 (0.31-2.83)	0.79	0.75 (0.091-6.12)
	≥2 sites vs. No	0.65	0.85 (0.42-1.73)	0.45	1.47 (0.54-3.95)
NED at start of TRO	Yes vs. No	**0.028**	0.50 (0.27-0.93)	*0.094*	0.50 (0.27-0.93)
Response to ifosfamide-containing therapy	SD/PD vs. PR/CR	0.71	1.14 (0.57-2.27)	0.77	0.88 (0.38-2.05)
Systemic induction therapy	AI vs. No	0.51	1.30 (0.60-2.82)	0.16	2.85 (0.66-12.32)
	Others vs. No	0.71	1.20 (0.46-3.15)	0.13	3.33 (0.69-16.05)
Response to induction therapy	SD vs. PR/CR	0.13	1.75 (0.86-3.59)	0.68	0.84 (0.37-1.91)
Prior systemic therapies	≤1 vs. >1	0.67	0.87 (0.46-1.65)	0.82	1.11 (0.47-2.63)
LT during TRO	Yes vs. No	0.59	1.21 (0.60-2.45)	0.53	0.71 (0.24-2.06)
Dose reduction or treatment interruption	Yes vs. No	*0.054*	0.57 (0.32-1.01)	0.84	1.08 (0.52-2.23)

UPS: Undifferentiated pleomorphic sarcoma, LPS: Liposarcoma, LMS: Leiomyosarcoma, SySa: Synovial sarcoma, TRO: Trofosfamide, NED: No radiological evidence of disease, SD: Stable disease, PD: Progressive disease, PR: Partial remission, CR: Complete remission, AI: Anthracycline + ifosfamide, LT: Local therapy

3. Multivariate analysis for event-free survival 2 (EFS2) after maintenance therapy with trofosfamide, multivariate analysis

		**EFS2**	
**Factor**	**Strata**	**p-value**	**HR (95% CI)**
Age (years)	≤60 vs. >60	0.50	2.14 (0.23-19.95)
Sex	Female vs. male	0.46	1.62 (0.45-5.88)
Histological subtype	Other histological subtypes vs. SySa	0.29	0.17 (0.0066-4.39)
NED at start of TRO	Yes vs. No	**0.0051**	0.060 (0.0084-0.43)
Subsequent therapy	Trabectedin vs. other therapies	0.47	0.48 (0.065-3.51)

TRO: Trofosfamide, NED: No radiological evidence of disease, SySa: Synovial sarcoma
